# Novel external reinforcement device for gastrointestinal anastomosis in an experimental study

**DOI:** 10.1186/s12893-023-02027-1

**Published:** 2023-05-11

**Authors:** Hiro Hasegawa, Nobuyoshi Takeshita, Woogi Hyon, Suong-Hyu Hyon, Masaaki Ito

**Affiliations:** 1 Department of Colorectal Surgery, National Cancer Center Hospital East, 6-5-1 Kashiwanoha, Kashiwa, Chiba 277-8577 Japan; 2 Surgical Device Innovation Office, NEXT medical device innovation center, National Cancer Center Hospital East, Kashiwa, Japan; 3 BMG Incorporated, Kyoto, Japan

**Keywords:** Anastomotic leakage, Anastomosis, Colorectal surgery, Reinforcement, Sealant

## Abstract

**Background:**

Anastomotic leakage has been reported to occur when the load on the anastomotic site exceeds the resistance created by sutures, staples, and early scars. It may be possible to avoid anastomotic leakage by covering and reinforcing the anastomotic site with a biocompatible material. The aim of this study was to evaluate the safety and feasibility of a novel external reinforcement device for gastrointestinal anastomosis in an experimental model.

**Methods:**

A single pig was used in this non-survival study, and end-to-end anastomoses were created in six small bowel loops by a single-stapling technique using a circular stapler. Three of the six anastomoses were covered with a novel external reinforcement device. Air was injected, a pressure test of each anastomosis was performed, and the bursting pressure was measured.

**Results:**

Reinforcement of the anastomotic site with the device was successfully performed in all anastomoses. The bursting pressure was 76.1 ± 5.7 mmHg in the control group, and 126.8 ± 6.8 mmHg in the device group, respectively. The bursting pressure in the device group was significantly higher than that in the control group (*p* = 0.0006).

**Conclusions:**

The novel external reinforcement device was safe and feasible for reinforcing the anastomoses in the experimental model.

## Background

Surgical resection is an important treatment option for patients with gastrointestinal disorders. Although gastrointestinal anastomosis after resection is a common surgical procedure, anastomotic leakage (AL) remains the most serious complication of gastrointestinal surgery. In rectal surgery, AL has been reported to occur in 6–14% of cases [1–4] and is associated with increased mortality and morbidity [3, 4], leading to longer length of hospital stay and increased medical costs [3]. In addition, some reports suggest that AL negatively impacts long-term survival in patients with cancer [5].

There are many local and systemic factors that may cause AL [6, 7]; although the risk of AL is multifactorial, the stability of the anastomoses has been shown to be one of the most important factors [6, 8, 9]. Thompson et al. reported that the strength of the anastomosis was lost during the first few days after surgery, and AL occurred when the load on the anastomotic site exceeded the durability of the sutures or staples and early scars [6]. Therefore, by reinforcing the anastomosis, AL may be avoided. In recent years, various materials have been proposed [10–12], some of which have been clinically applied to reduce AL [11, 12]. However, these products have various problems, such as reduced safety and strength, and their efficacy has not yet been demonstrated [10–12].

To solve these problems, we developed a novel bioabsorbable device consisting of two layers, a sheet with a physical reinforcing effect, and a sealant with adhesive strength. The aim of this study was to evaluate the safety and feasibility of this novel external reinforcement device for gastrointestinal anastomosis in an experimental model.

## Methods

### Animal

The experimental animal was purchased from KAC Company Limited (Kyoto, Japan). A single pig (domestic, female, crossbred with Large Yorkshire and Landrace, weight 49.6 kg, 3 months of age) was used in this non-survival study. The study protocol was approved by the Committee for Ethics of Animal Experimentation of the National Cancer Center (K17–012). The experiment was performed according to the Animal Research: Reporting of In Vivo Experiments (ARRIVE) guidelines [13] and the Guidelines for Animal Experiments (Science Council of Japan: Guidelines for Proper Conduct of Animal Experiments, 2006).

### Novel external reinforcement device

**Fig. 1:**
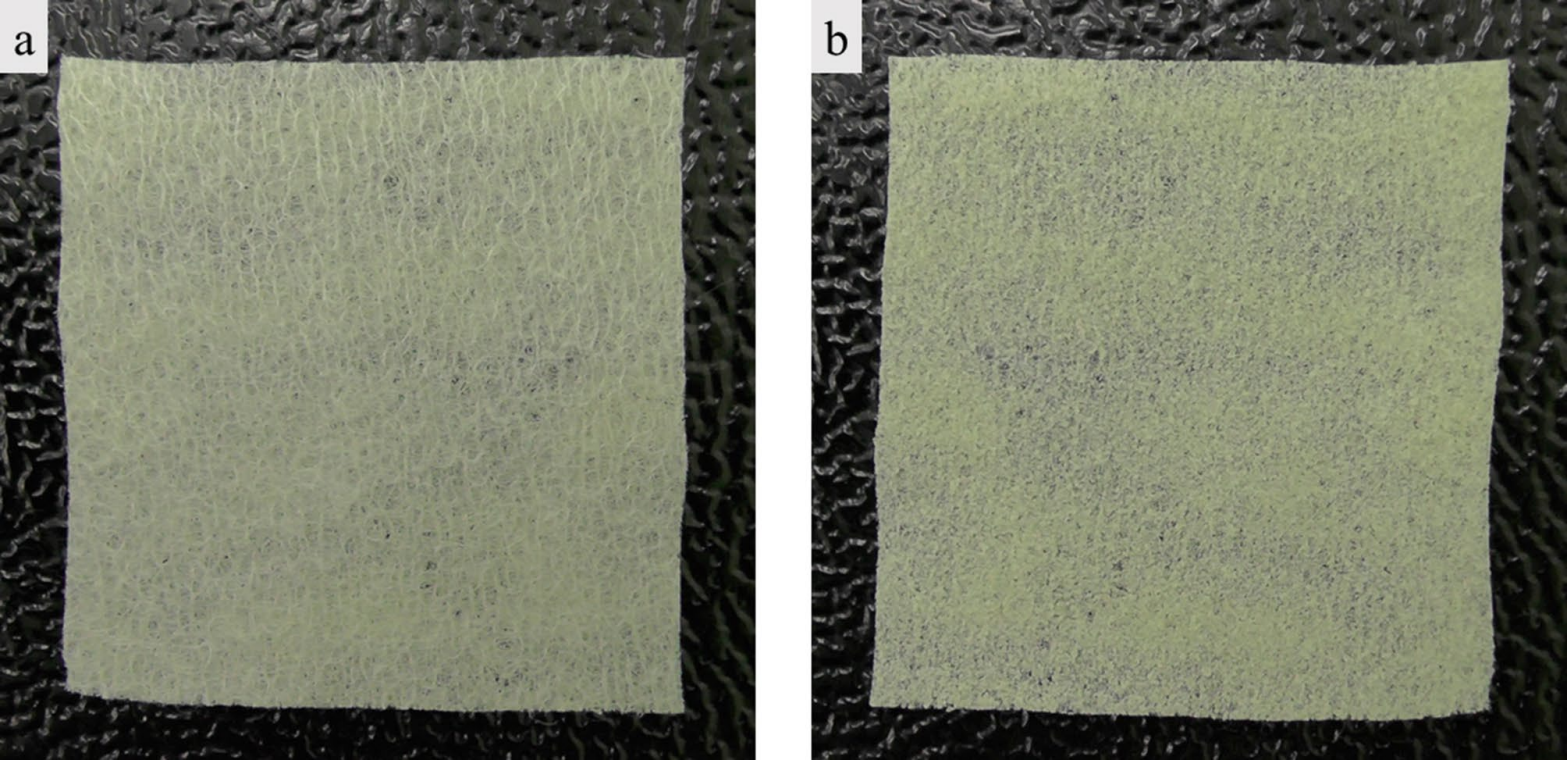
Novel external reinforcement device. **a** The outer sheet consists of polyglycolic acid. **b** The inner sealant consists of LYDEX®

We developed a novel external reinforcement device for gastrointestinal anastomosis in conjunction with BMG Incorporated (Kyoto, Japan). The device consists of two bioabsorbable layers, a sheet with a physical reinforcing effect, and a sealant with adhesive strength (Fig. 1). The outer sheet consists of polyglycolic acid (PGA), and the inner sealant consisting of LYDEX® [14–19].

**Fig. 2:**
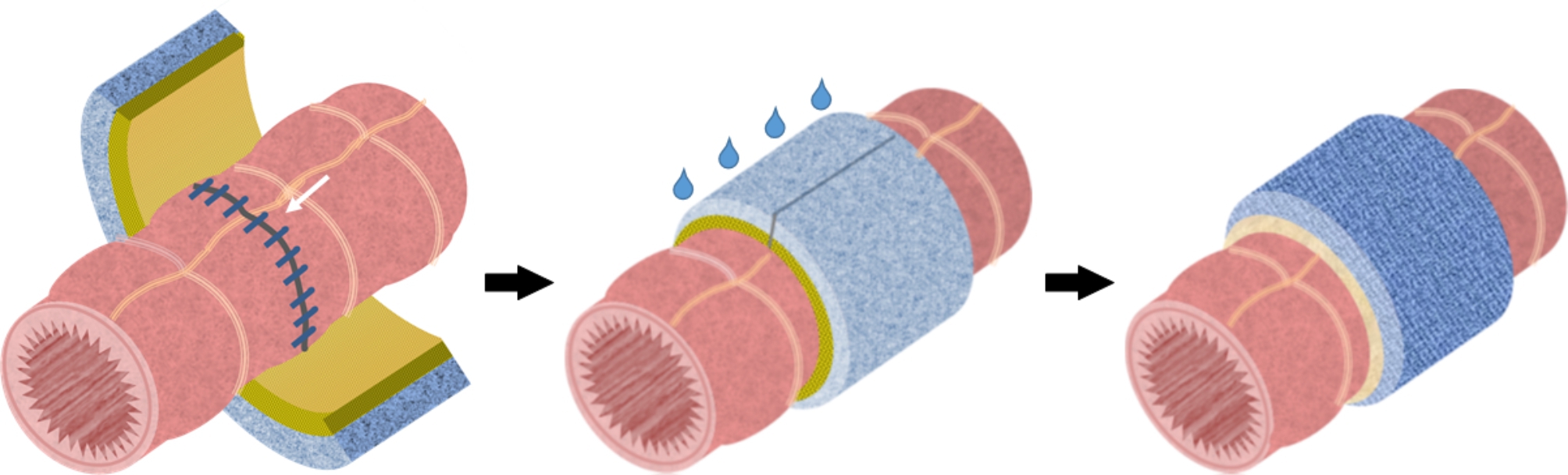
Reinforcement of the anastomosis using the device. White arrow indicates the anastomosis. The device is wrapped around the gastrointestinal tract. Saline is infiltrated from the outside to the gel LYDEX®. The device is adhered to the gastrointestinal tract

The device is stable at room temperature and does not require preparation before use. The device can be cut into small pieces and placed according to the object size and orientation. The device was used as follows: it was wrapped around the gastrointestinal tract with the inner sealant LYDEX® side inside, to cover the anastomotic site (Fig. 2). Saline was infiltrated from the outside to the gel LYDEX® and adhered to the gastrointestinal tract (Fig. 2).

### Study protocol

After the pig was premedicated with an intramuscular injection of ketamine (10 mg/kg) and xylazine (2 mg/kg), general anesthesia was induced with 5% isoflurane. The airway was secured by endotracheal intubation, and general anesthesia was maintained with 1–3% isoflurane.

**Fig. 3:**
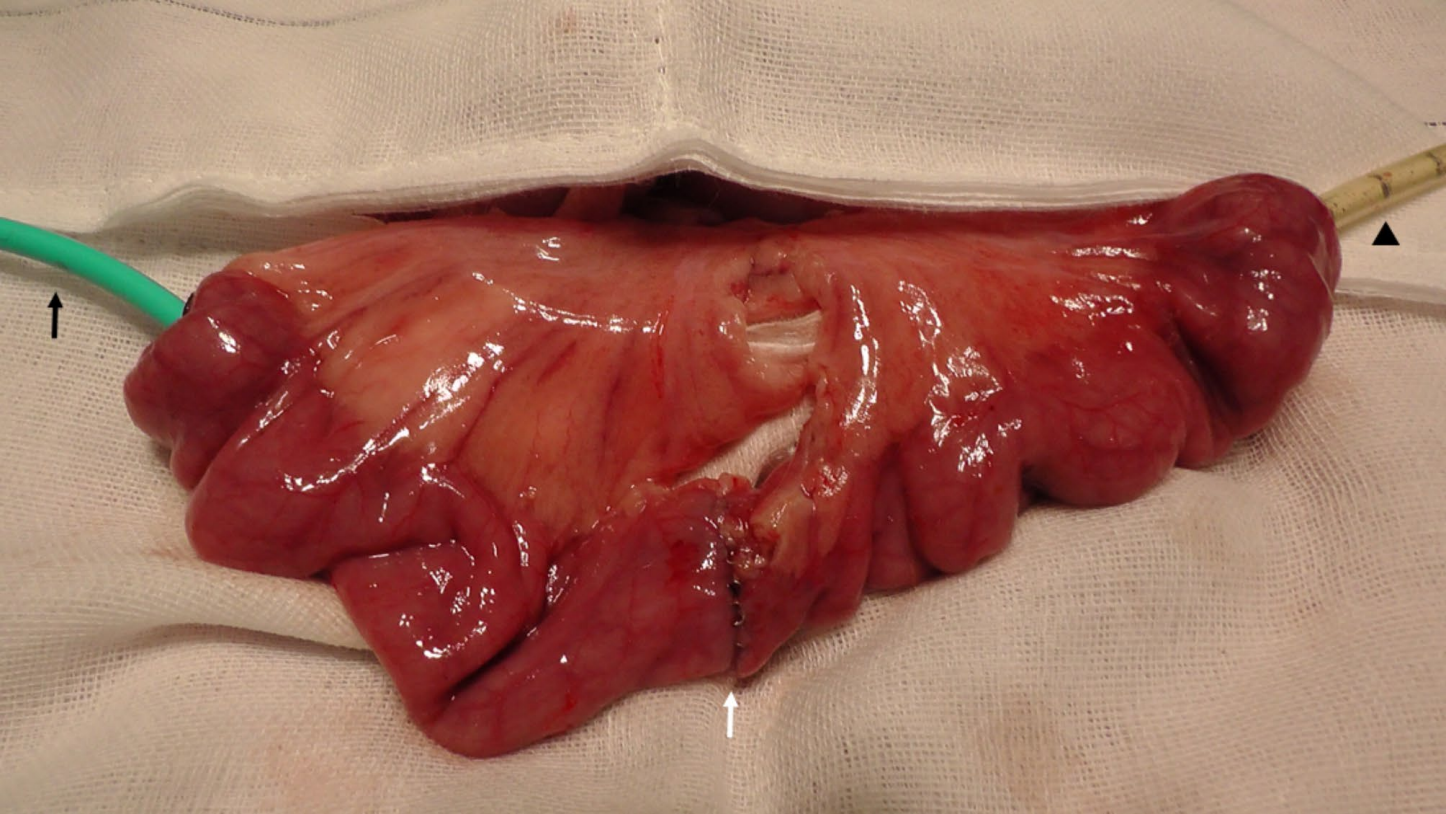
Bursting model. Black arrow head indicates the manometry catheter. Black arrow indicates a 10-Fr silicone tube. White arrow indicates the anastomosis

A standard ventral midline laparotomy was performed under general anesthesia. End-to-end anastomoses were created in the six small bowel loops by a single-stapling technique using a 21-mm diameter circular stapler (PROXIMATE ILS Straight Intraluminal Stapler; Ethicon Endo-Surgery Incorporated, Cincinnati, OH, USA). After completion of the anastomosis, the manometry catheter with one pressure-sensing channel (Star Medical Incorporated, Tokyo, Japan) and a 10-Fr silicone tube were inserted intraluminally from the oral and anal stumps, respectively (Fig. 3). The manometry catheter and the silicone tube were secured twice with a purse-string suture using a 3 − 0 silk suture, respectively. Three of the six anastomoses were covered with the device, while the remaining three were not. Air was manually injected through the tube at an arbitrary rate until the pressure started to build up, then manually at a rate of 2 ml/sec, and a pressure test for each anastomosis was performed. The bursting pressure was measured using analysis software (GMMS-100, Star Medical Incorporated, Tokyo, Japan), and the bursting site was recorded. Bursting was defined as a rapid decrease in the maximum pressure measured in real time using the manometry system and no increase in pressure despite continuous air injection. At the end of the procedure, the experimental animal was sacrificed by intravenous injection of a lethal dose of potassium chloride under deep general anesthesia.

### Statistical analysis

Statistical analyses were performed using JMP version 15.1.0 (SAS Institute, Cary, NC, USA). Values are reported as mean and standard deviation. For the comparison of bursting pressure, Student’s t-test was utilized. Statistical significance was set at *P* < 0.05.

## Results

All surgical procedures were uneventful. The establishment of the bursting model was systematically reproduced in all anastomoses. Reinforcement of the anastomotic site with the novel external coating device was successfully performed in all anastomoses.

**Fig. 4:**
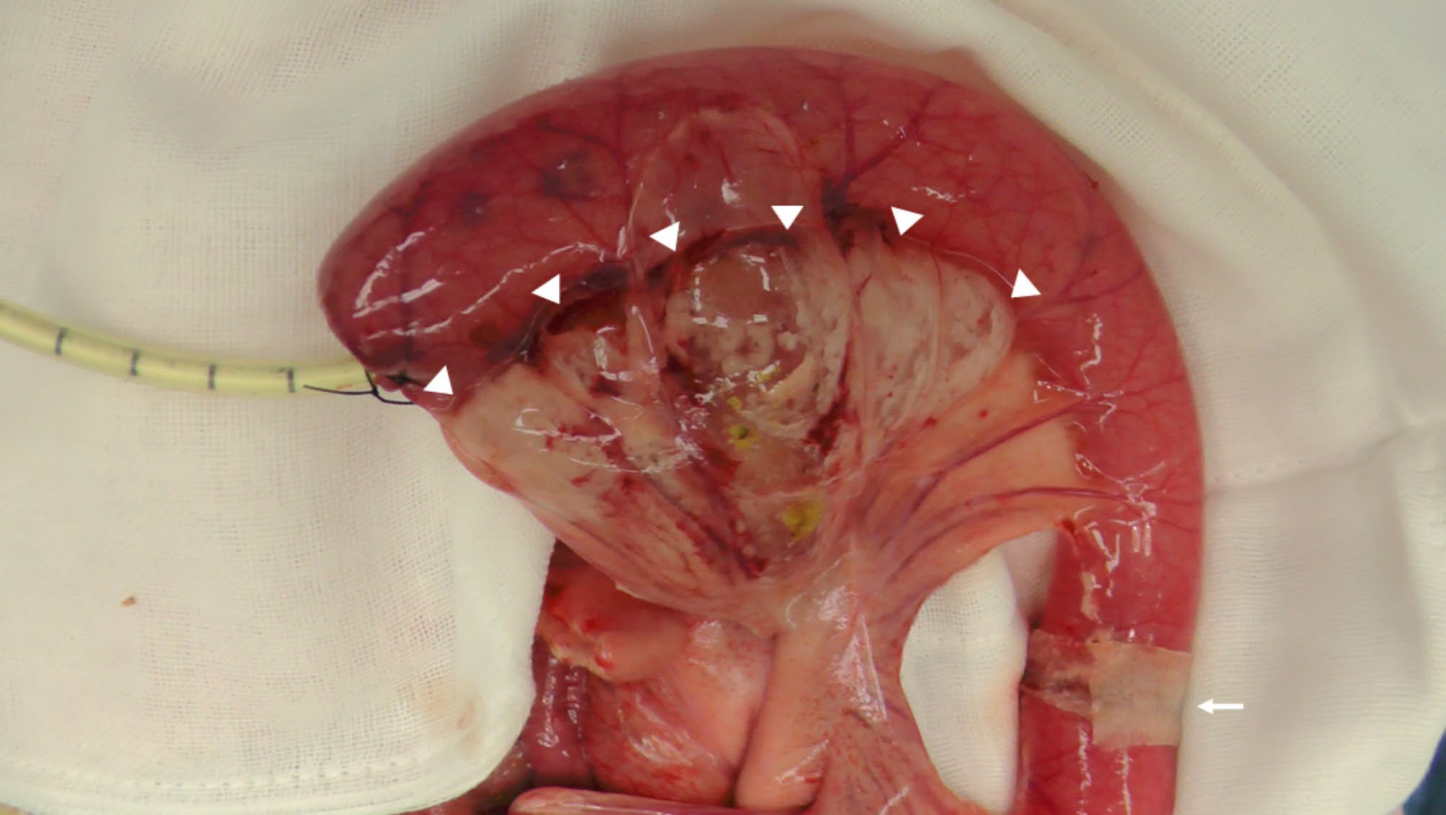
Perforation of the gastrointestinal tract. White arrow heads indicate the mesenteric emphysema. White arrow indicates the anastomosis covered with the device

**Fig. 5:**
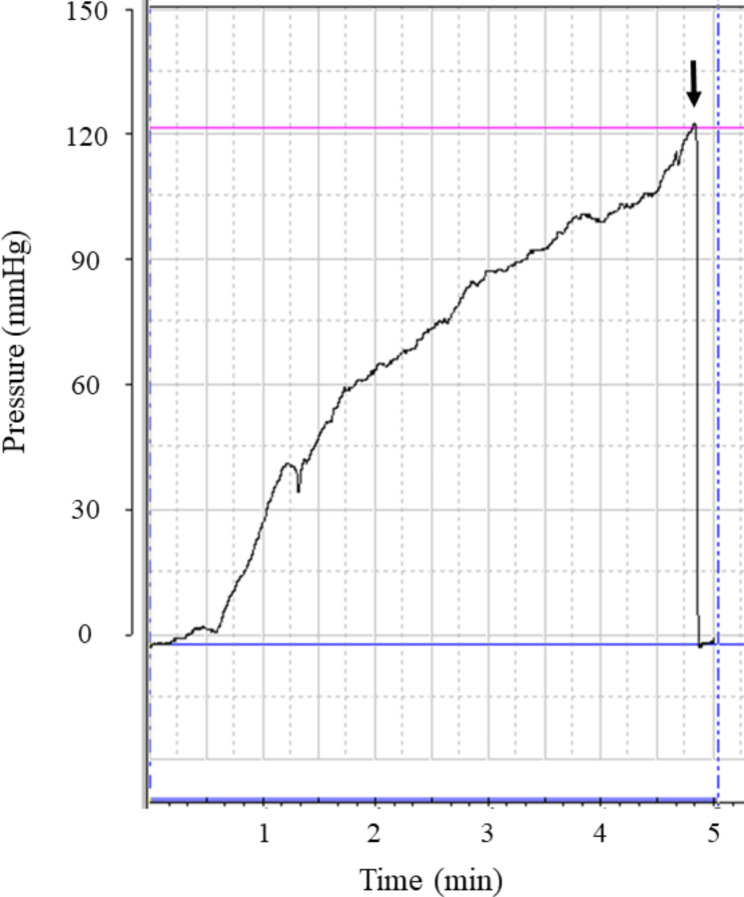
Bursting pressure. Black arrow indicates the bursting pressure

**Fig. 6:**
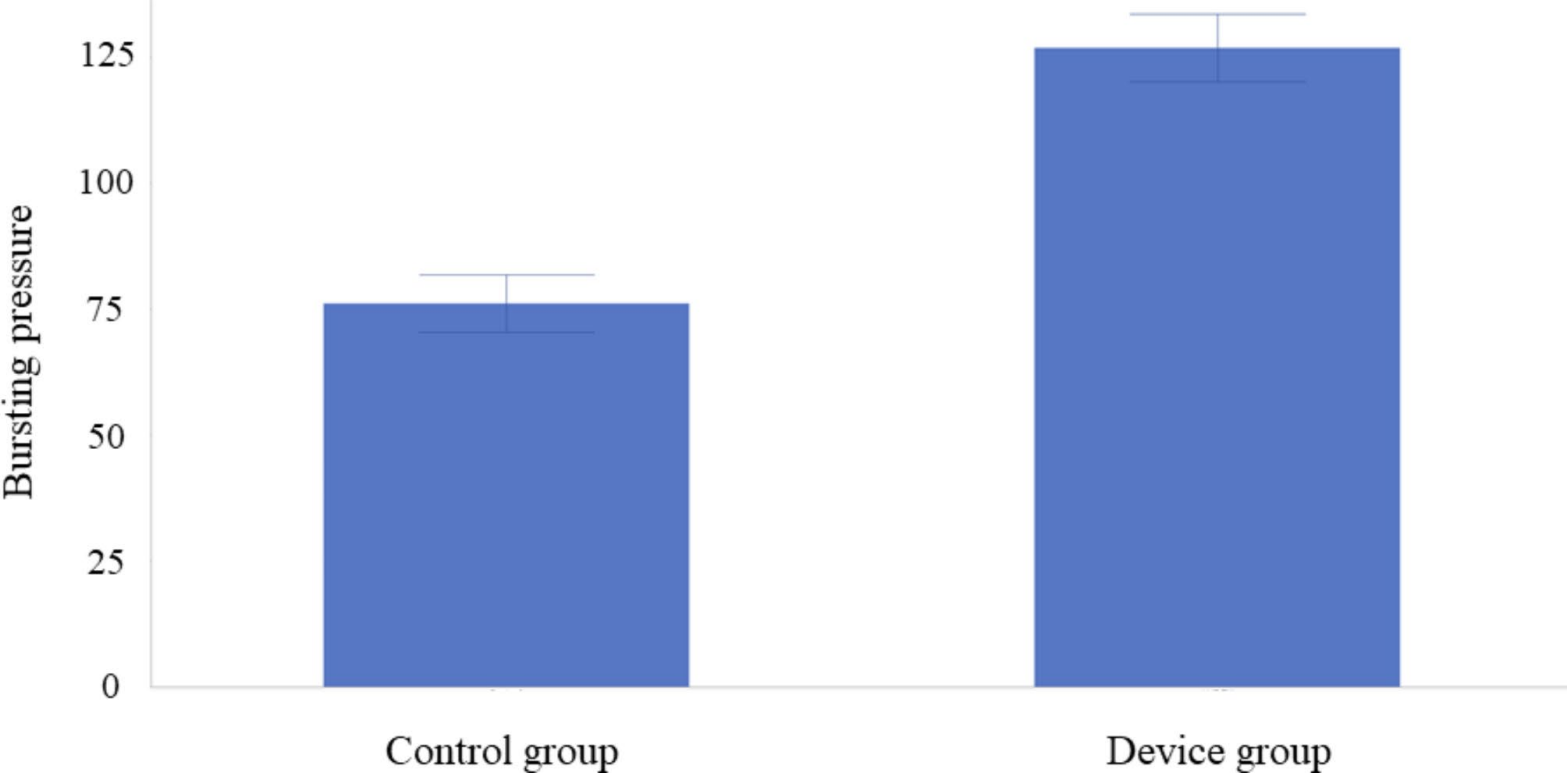
Comparison of the average bursting pressure between the two groups

In the control group (n = 3), the bursting pressure was 76.1 ± 5.7 mmHg, and air leakage was observed from the staple line in all anastomoses. The bursting pressure was 126.8 ± 6.8 mmHg in the device group (n = 3). In one of the three anastomoses, air leakage was observed from the staple line. In contrast, in two out of three anastomoses, perforation was observed from the gastrointestinal tract on the mesenteric side instead of the staple line, and mesenteric emphysema was observed (Fig. 4). One result of the bursting pressure in the device group obtained using the analysis software (GMMS-100, Star Medical Incorporated, Tokyo, Japan) is shown in Fig. 5. The bursting pressure in the device group was significantly higher than that in the control group (*p* = 0.0006) (Fig. 6).

## Discussion

Reinforcement of the anastomotic site with the device was successfully performed in all anastomoses, and the bursting pressure in the device group was significantly higher than that in the control group.

AL has been reported to occur when the load on the anastomotic site exceeds the physical strength of sutures or staples [6]. Gastrointestinal anastomosis loses much of its durability within the first few days after surgery [6]. The bursting pressure of the anastomosis is lowest at 2 to 3 days after surgery, and it is 50% in the small intestine and 35–75% in the large intestine, compared with that in the normal intestine [6]. Finally, it recovers to 100% 7 days postoperatively [6]. Therefore, it may be possible to avoid AL by covering and reinforcing the anastomotic site while the anastomotic site is fragile. In recent years, there have been reports of external coating of the anastomotic site with various materials [10–12]. It has been reported that fibrin glue [11] and hyaluronic acid/carboxymethylcellulose [12] have been clinically applied in colorectal surgery.

Fibrin glue is a sealant that contains human blood-derived materials such as fibrinogen and utilizes the mechanism of blood coagulation. It has the potential to cause blood-borne infections, such as parvovirus infection or hepatitis. In addition, there is a drawback that preparation at the time of use is required. Huh et al. [11] reported the effect of fibrin glue in laparoscopic surgery for rectal cancer. In the fibrin glue group, fibrin glue was applied to the anastomosis. There was no significant difference in the rate of AL between the fibrin glue and control groups (6/104, 5.8% vs. 13/119, 10.9%; *p* = 0.17).

Hyaluronic acid/carboxymethylcellulose (Seprafilm®) is a bioabsorbable film that is not made from human- or animal-derived components. Beck et al. [12] reported the results of a prospective, randomized, multicenter, controlled study to demonstrate the safety and effectiveness of Seprafilm® in colorectal surgery. In the Seprafilm® group, the anastomosis was wrapped with Seprafilm® in 289 of 882 cases. The rates of AL-related complications, including AL, anastomotic fistula, peritonitis, abdominopelvic abscess, and sepsis, were significantly higher in the Seprafilm® group than in the control group (39/289, 13.5% vs. 46/909, 5.1%; *p* < 0.001).

**Fig. 7:**
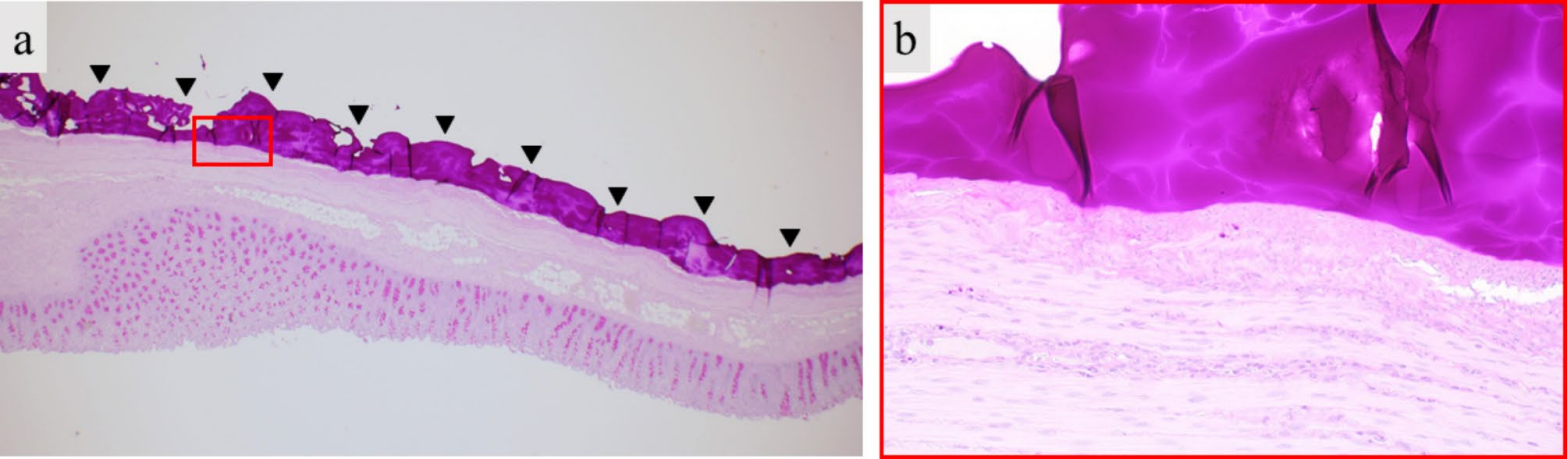
Histological findings (periodic acid-Schiff stain). Black arrow head indicates LYDEX®. **a** High ability to adhere to the porcine gastrointestinal tract. **b** Enlarged view of the red rectangle in a

Therefore, these products have various problems, such as reduced safety and strength, and their efficacy has not yet been demonstrated [10–12]. To solve these problems, we developed a novel external reinforcement device, which is an external bioabsorbable reinforcement device for gastrointestinal anastomosis composed of two layers, a sheet with a physical reinforcing effect, and a sealant with adhesive strength. To the best of our knowledge, there are no devices with such a concept. PGA and LYDEX® are not made from human- and animal-derived materials, so they are extremely safe without the risk of infection. PGA is widely used in clinical practice as a medical material for bioabsorbable polymers. By processing the outer sheet of PGA into a three-dimensional (3D) mesh, it not only has high strength, but also high flexibility to follow bowel peristalsis. From the viewpoint of strength and wound healing, a sheet made by processing a polymer compound into a 3D mesh is suitable for this application [20]. The inner sealant, LYDEX®, consists of aldehyde dextran, which is a high-molecular-weight pharmaceutical raw material, and ε-poly (L-lysine), which is a food additive [14–19]. LYDEX® has the following features. First, it has a higher adhesive strength than fibrin glue, which is widely used as a sealant. In an in vitro tensile strength test, a mesh sheet made with LYDEX® (n = 8) had significantly higher tensile strength than that made with fibrin glue (n = 8) (3.3 ± 0.59 N vs. 0.33 ± 0.14 N; *p* < 0.01). Second, since LYDEX® instantly gels upon contact with water, a high degree of airtightness can be obtained by 3D bonding to the object. Previous experiments have histologically shown a high ability to adhere to the porcine gastrointestinal tract (Fig. 7). Third, LYDEX® has a strong antibacterial effect, so it is suitable for gastrointestinal surgery, which is classified as a clean-contaminated or contaminated surgery [21]. In this study, the device was safely applied to the anastomotic site without adverse events, and showed a significant reinforcing effect. The anastomotic site was wrapped with the device during the laparotomy. However, the number of laparoscopic surgeries is increasing, and it is necessary to develop a method to easily apply the device to the anastomotic site to improve these devices for practical applications.

There are some limitations to this study. First, this study was conducted in accordance with the 3Rs principles [22]. Only one pig was used, and it is not clear whether the device can be extrapolated to other pigs. Furthermore, although all anastomoses were created in the small bowel, there may be a difference in bursting pressure depending on the region of the bowel, and it is not clear whether the results can be extrapolated to other region of the bowel, such as the rectum or colon. However, we believe that this limitation is minimal, because all procedure were performed in the same manner, and reinforcement of the anastomotic site with the device was successfully performed in all anastomoses. This study is a pilot study, and we believe that using the small bowel from the viewpoint of confirming mechanical breakdown is not a major limitation. Further studies are needed to assess the feasibility of the use of this device in other regions of the bowel. Second, this was a non-survival study. Regarding safety, PGA is widely used in clinical practice as a medical material for bioabsorbable polymers. It has previously been reported that PGA can induce the local inflammatory response following implantation in vitro and in vivo [23, 24]. However, evaluations of the cytotoxicity of PGA have reported that PGA has very low cytotoxicity [25, 26]. A histological evaluation of the local inflammation of LYDEX® has also been reported [16], in which the appearance of macrophages was observed up to 2 weeks after implantation of LYDEX®, but gradually decreased thereafter, almost disappearing after 4 weeks. The evaluation of the cytotoxicity of LYDEX® showed that LYDEX® and its degradation products showed very low cytotoxicity [14, 15]. A clinical trial using LYDEX® for other uses has already been conducted, and its safety has been reported [27, 28]. However, this study does not show whether the device adheres to the anastomotic site in the long term or increases the risk of AL; therefore, further studies are needed to assess the long-term effects of this device. Third, PGA has been reported to be hydrolyzed to glycolic acid, and metabolized into water and carbon dioxide within 15 weeks [29]. It has further been reported that LYDEX® can form a hydrogel, and that the weight of the LYDEX® hydrogel decreases to 0 after 2 weeks in the saline immersion test [17]. Since the test was conducted under stricter conditions than those encountered in vivo, it is believed that the degradation of LYDEX® will be slower in vivo. However, the hydrolysis time can be controlled by adjusting the composition in LYDEX®; therefore, we believe that this limitation is solvable. Further studies are needed to assess the durability of this device.

## Conclusions

The novel external reinforcement device was safe and feasible for reinforcing the anastomoses in the experimental model.

## Data Availability

The datasets used and/or analyzed during the current study are available from the corresponding author on reasonable request.

## Abbreviations

AL: anastomotic leakage
PGA: polyglycolic acid
3D: three-dimensional

## References

[1] Pommergaard HC, Gessler B, Burcharth J, Angenete E, Haglind E, Rosenberg J (2014). Preoperative risk factors for anastomotic leakage after resection for colorectal cancer: a systematic review and meta-analysis. Colorectal Dis.

[2] Qu H, Liu Y, Bi DS (2015). Clinical risk factors for anastomotic leakage after laparoscopic anterior resection for rectal cancer: a systematic review and meta-analysis. Surg Endosc.

[3] Kang CY, Halabi WJ, Chaudhry OO, Nguyen V, Pigazzi A, Carmichael JC (2013). Risk factors for anastomotic leakage after anterior resection for rectal cancer. JAMA Surg.

[4] Snijders HS, Wouters MW, van Leersum NJ, Kolfschoten NE, Henneman D, de Vries AC (2012). Meta-analysis of the risk for anastomotic leakage, the postoperative mortality caused by leakage in relation to the overall postoperative mortality. Eur J Surg Oncol.

[5] Mirnezami A, Mirnezami R, Chandrakumaran K, Sasapu K, Sagar P, Finan P (2011). Increased local recurrence and reduced survival from colorectal cancer following anastomotic leak: systematic review and meta-analysis. Ann Surg.

[6] Thompson SK, Chang EY, Jobe BA (2006). Clinical review: healing in gastrointestinal anastomoses, part I. Microsurgery.

[7] Kingham TP, Pachter HL (2009). Colonic anastomotic leak: risk factors, diagnosis, and treatment. J Am Coll Surg.

[8] Marescaux JF, Aprahamian M, Mutter D, Loza E, Wilhelm M, Sonzini P (1991). Prevention of anastomosis leakage: an artificial connective tissue. Br J Surg.

[9] Hoeppner J, Crnogorac V, Marjanovic G, Jüttner E, Keck T, Weiser HF (2009). Small intestinal submucosa for reinforcement of colonic anastomosis. Int J Colorectal Dis.

[10] Pommergaard HC, Achiam MP, Rosenberg J (2012). External coating of colonic anastomoses: a systematic review. Int J Colorectal Dis.

[11] Huh JW, Kim HR, Kim YJ (2010). Anastomotic leakage after laparoscopic resection of rectal cancer: the impact of fibrin glue. Am J Surg.

[12] Beck DE, Cohen Z, Fleshman JW, Kaufman HS, van Goor H, Wolff BG (2003). A prospective, randomized, multicenter, controlled study of the safety of Seprafilm adhesion barrier in abdominopelvic surgery of the intestine. Dis Colon Rectum.

[13] Kilkenny C, Browne WJ, Cuthill IC, Emerson M, Altman DG (2010). Improving bioscience research reporting: the ARRIVE guidelines for reporting animal research. PLOS Biol.

[14] Hyon SH, Nakajima N, Sugai H, Matsumura K (2014). Low cytotoxic tissue adhesive based on oxidized dextran and epsilon-poly-L-lysine. J Biomed Mater Res A.

[15] Matsumura K, Nakajima N, Sugai H, Hyon SH (2014). Self-degradation of tissue adhesive based on oxidized dextran and poly-L-lysine. Carbohydr Polym.

[16] Takai F, Takeda T, Yamazaki K, Ikeda T, Hyon SH, Minatoya K (2020). Management of retrosternal adhesion after median sternotomy by controlling degradation speed of a dextran and ε-poly (L-lysine)-based biocompatible glue. Gen Thorac Cardiovasc Surg.

[17] Hyon W, Hyon SH, Matsumura K (2022). Evaluation of the optimal dose for maximizing the anti-adhesion performance of a self-degradable dextran-based material. Carbohydr Polym Technol.

[18] Nonsuwan P, Matsugami A, Hayashi F, Hyon SH, Matsumura K (2019). Controlling the degradation of an oxidized dextran-based hydrogel independent of the mechanical properties. Carbohydr Polym.

[19] Araki M, Tao H, Nakajima N, Sugai H, Sato T, Hyon SH (2007). Development of new biodegradable hydrogel glue for preventing alveolar air leakage. J Thorac Cardiovasc Surg.

[20] Takeshita N, Ho KY (2016). Endoscopic closure for full-thickness gastrointestinal defects: available applications and emerging innovations. Clin Endosc.

[21] Lee JH, Kim HL, Lee MH, Taguchi H, Hyon SH, Park JC (2011). Antimicrobial effect of medical adhesive composed of aldehyded dextran and ε-poly(L-lysine). J Microbiol Biotechnol.

[22] Russell WMS, Burch RL (1959). The principles of humane experimental technique.

[23] Ceonzo K, Gaynor A, Shaffer L, Kojima K, Vacanti CA, Stahl GL (2006). Polyglycolic acid-induced inflammation: role of hydrolysis and resulting complement activation. Tissue Eng.

[24] Yang H, Dong Z, Wang H, Liu Z, Sun W, Wang K (2022). Efficacy of oxidized regenerated cellulose combined with fibrin glue in reducing pulmonary air leakage after segmentectomy in a porcine lung model. Front Bioeng Biotechnol.

[25] Hajiali H, Shahgasempour S, Naimi-Jamal MR, Peirovi H (2011). Electrospun PGA/gelatin nanofibrous scaffolds and their potential application in vascular tissue engineering. Int J Nanomedicine.

[26] Fu S, Zhang P (2019). Surface modification of polylactic acid (PLA) and polyglycolic acid (PGA) monofilaments via the cold plasma method for acupoint catgut-embedding therapy applications. Text Res J.

[27] Park JS, Bang BW, Hong SJ, Lee E, Kwon KS, Kim HK (2019). Efficacy of a novel hemostatic adhesive powder in patients with refractory upper gastrointestinal bleeding: a pilot study. Endoscopy.

[28] Shin J, Cha B, Park JS, Ko W, Kwon KS, Lee JW (2021). Efficacy of a novel hemostatic adhesive powder in patients with upper gastrointestinal tumor bleeding. BMC Gastroenterol.

[29] Kanai E, Matsutani N, Aso T, Yamamoto Y, Sakai T (2020). Long-term effects of pleural defect repair using sheet materials in a canine model. Gen Thorac Cardiovasc Surg.

